# Hypertension in Women: A South-Asian Perspective

**DOI:** 10.3389/fcvm.2022.880374

**Published:** 2022-08-10

**Authors:** Fatima Farrukh, Amin Abbasi, Misbah Jawed, Aysha Almas, Tazeen Jafar, Salim S. Virani, Zainab Samad

**Affiliations:** ^1^Medical College, Aga Khan University, Karachi, Pakistan; ^2^Medical College, Ziauddin University, Karachi, Pakistan; ^3^Baylor College of Medicine, Houston, TX, United States; ^4^Department of Medicine, Duke University, Durham, NC, United States

**Keywords:** gender, hypertension, sex-specific, South Asia, women, gender-specific differences

## Abstract

**Introduction:**

Hypertension is an important contributor to cardiovascular disease related morbidity and mortality. Despite the magnitude of its negative impact on cardiovascular outcomes, treatment and control of hypertension remain suboptimal in both men and women.

**Materials and Methods:**

Numerous databases, i.e., PubMed, ScienceDirect, etc., were searched using keywords to identify relevant studies to our narrative review. The findings from the most pertinent articles were summarized and integrated into our narrative review on hypertension in women.

**Results:**

The pathophysiology of essential hypertension is still being delineated in both men and women; there are multiple sex specific factors in association with the development of hypertension in women, including age, combined oral contraceptives (COCs), polycystic ovarian syndrome (PCOS), preeclampsia, etc. There are several sex specific considerations in antihypertensives drug choices.

**Discussion:**

Despite the magnitude of its negative impact on cardiovascular outcomes, treatment and control of hypertension remain suboptimal in women. Medical treatment and adherence is uniquely challenging for South Asian women due to a variety of socio-cultural-economic factors. Further research is warranted to identify optimal sex-specific treatment options that will improve the control of hypertension and decrease the risk of subsequent cardiovascular disease in both genders.

## Introduction

Hypertension is an important contributor to cardiovascular disease related morbidity and mortality. With at least 7.6 million deaths per year worldwide attributed to hypertension, it is recognized as a global public health problem (1). Despite the magnitude of its negative impact on cardiovascular outcomes, treatment and control of hypertension remain suboptimal in both men and women. There are multiple sex-specific factors associated with hypertension in women. Efforts to control hypertension, including hypertension control programs and risk assessment models by the CDC and WHO do not consider gender specifically (2, 3). There are differences in men and women regarding hypertension prevalence, risk factors, pathophysiology, complications and treatment. Here we provide a narrative review of the differences between men and women regarding hypertension prevalence, risk factors, pathophysiology, complications and treatment.

## Epidemiology

Globally, over 1.13 billion people currently suffer from hypertension (4). Since 1990, the prevalence of hypertension has doubled with most of the increase happening in low-income and middle-income regions (5). From 2000 to 2010, there was an increase in hypertension prevalence of 5.2% over 10 years. A recent cross-sectional study with a pooled data-set from 1.1 million adults from 44 low middle income countries (LMICs) found hypertension prevalence in middle income countries to be 17.5% (6).

The estimated global prevalence of hypertension was 24.1% for men and 20.1% for women in 2015 (7). Research studies have found men to have significantly higher rates of hypertension compared with women in high-income countries (44 versus 32%, respectively), and women to have slightly higher rates of hypertension compared with men in LMICs [39% for women versus 37% for men (8)]. Studies predict a 13% increase in the prevalence of hypertension in women and a 9% increase in men by 2025; 483.5 million women had hypertension in 2000 which is estimated to increase to 793.3 million in 2025 (9).

South Asian nations represent 24.9% of the world population, and they are undergoing a rapid epidemiological transition with significant rates of hypertension in the different countries (10). In an Indian nationally representative study of 1.3 million adults, carried out between 2012 and 2014, the prevalence of hypertension in women and men was 23.6 and 27.4%, respectively (11). Non-Communicable Diseases (NCD) Survey of Pakistan (2016) found 36.8% of men diagnosed with Stage I hypertension compared to 29.3% of women (12). In a Sri Lankan community-based national survey in 2014, the prevalence of hypertension was 23.4% in men and 23.8% in women; their results revealed nearly one-third of the Sri Lankan population to be hypertensive (13). The national survey for non-communicable disease risk factors and mental health using WHO STEPS approach in Bhutan found 33.6% men hypertensive and 32% women in 2014 (14). In the Bangladesh Demographic and Health Survey 2017–2018, the prevalence of hypertension was slightly higher in women compared with men, 28 and 26%, respectively. According to the Maldives Demographic and Health Survey 2015–2017, a mere 4% of women and 2% of men said they had been diagnosed with hypertension by health professionals (15). In Afghanistan, due to unfortunate circumstances, it has been difficult to make accurate estimates of NCD’s prevalence, including hypertension. However, a provincial cross-sectional study in 2015 using WHO STEP-wise approach found the prevalence of hypertension among adult Kabul citizens to 51.1% in females and 48.9% in males (16).

Table 1 displays the prevalence of hypertension in South-Asian countries according to the most recent available data. It is important to recognize that a large proportion of hypertension remains undiagnosed. One in six United States adults, approximately 11 million, have undiagnosed hypertension; the rates are expected to be higher in LMICs due to lower accessibility to healthcare (17).

**TABLE 1:** Prevalence of hypertension in South Asian countries according to most recent available data.

Country	Gender	Prevalence (%)	Year of the latest survey data
Pakistan	Male	36.8	2016
	Female	29.3	
India	Male	27.4	2018
	Female	23.6	
Bangladesh	Male	26.0	2017
	Female	28.0	
Nepal	Male	24.3	2016
	Female	16.9	
Bhutan	Male	33.6	2014
	Female	32.0	
Sri Lanka	Male	6.0	2014
	Female	10.3	
Afghanistan	Male	48.9	2015
	Female	51.1	
Maldives	Male	2	2017
	Female	4	

Hypertension does seem to follow a more aggressive path in the South-Asian population (18, 19). Their chances of being hypertensive at a younger age are nearly three times greater than European whites (20, 21). However, evidence over whether hypertension is more common in South-Asians is ambiguous (22, 23). A large population cohort study found the incidence of diagnosed hypertension to be highest in South Asians compared with Chinese and white patients (24). Another study reports higher diastolic blood pressure in South Asian men than the general population with no differences among women (22).

## Risk Factors for Hypertension in Women

**FIGURE 1:**
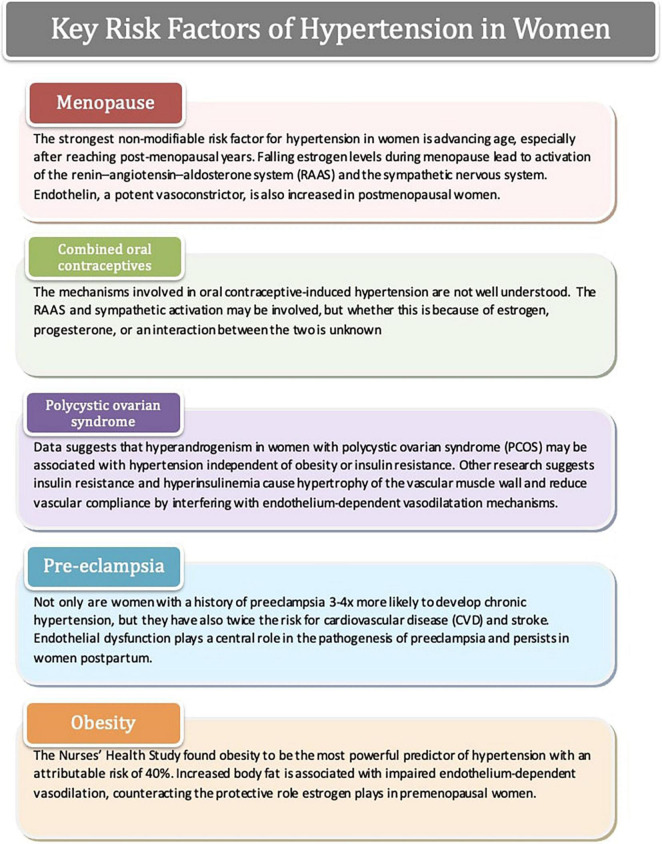
Key risk factors of hypertension in women.

Hypertension, underdiagnosed and subsequently undertreated, is an ever-increasing problem for women, with a lifetime risk of developing hypertension to be approximately 90% (25–27). Whilst the mechanism of essential hypertension remains unknown in both men and women, recent studies have proposed multiple factors in association with the development of hypertension in women, including age, combined oral contraceptives (COCs), polycystic ovarian syndrome (PCOS), preeclampsia, etc. (Figure 1). Table 2 displays the pathophysiology of hypertension in men and women.

**TABLE 2:** Pathophysiology of hypertension in men and women.

	Men ♂	Women ♀
• Atherosclerosis	++	+
• Genetic predisposition	+	+
• Environmental factors (smoking, etc.)	+	+
• Lack of endogenous estrogen	++ (young men)	−
• **Post-menopause**		
° Decreased estrogen	−	++
° Increased endothelin	−	++
° Renin activity	−	++
• **PCOS:**		
° Insulin resistance	−	++
° Hyperandrogenism	−	++

*+, Contributes to the pathophysiology of hypertension.*

*−, Does not contribute to the pathophysiology of hypertension.*

### Age/Menopause

The prevalence of hypertension is lower in premenopausal women compared with men of similar age (28). This difference is particularly pronounced in early adulthood. One study found that among 18–29-year-old White adults, just 1.5% of women but over 5% of men reported hypertension (29). But after menopause, occurring at an average age of 51, the American Heart Association (AHA) reports a steep rise in hypertension rates in women. From 45 to 64 years of age, the percentages of men and women with hypertension are similar and, after that, 5.3 and 11.8% more women have high blood pressure than men in ages 65–74 and 75+, respectively (30). Menopause is associated with a two-fold increase in risk of hypertension, with a prevalence of 75% in postmenopausal women in the United States (31). This pattern is further corroborated by the differences in the life course trajectory of systolic blood pressure (SBP) in men and women. SBP ranges are lower in women than men in early adulthood but experiences a steep rise after the midlife era so that by the seventh decade, men and women have similar average SBPs (32).

The strongest non-modifiable risk factor for hypertension in women is advancing age, especially after reaching post-menopausal years (33). Falling estrogen levels during menopause lead to the activation of the renin–angiotensin–aldosterone system (RAAS) and the sympathetic nervous system (34). RAAS is an important regulator of blood volume and systemic vascular resistance. In response to decreased perfusion, the kidney secretes renin, and angiotensin II is formed by a series of conversions. Angiotensin II raises blood pressure through vasoconstriction and aldosterone production. Renin activity has been shown to be higher in postmenopausal women compared to men and premenopausal women (35). Studies have further demonstrated that women who receive estrogen replacement therapy during menopause have significantly lower renin levels than those who do not receive hormone replacement (36).

Endothelin levels are increased in postmenopausal women (37). Endothelin is a potent vasoconstrictor that increases sodium reabsorption in the kidney and ultimately increases blood pressure (35). The mechanism behind increased endothelin levels in postmenopausal women is unclear; it may be mediated by angiotensin II or the altered androgen estrogen/testosterone ratio associated with menopause (38). Estradiol inhibits endothelin synthesis, thus, decreased estrogen levels after menopause leads to upregulation of endothelin production (35, 39).

Importantly, postmenopausal hormone replacement has not proved to be an effective preventive measure (40, 41). The effects of natural estrogen appear to be different from synthetic estrogen. Postmenopausal women not on hormone replacement therapy (HRT) have been shown to have increased levels of endothelin, however, studies have demonstrated that hormone replacement therapy with either micronized 17β-estradiol and dydrogesterone or conjugated equine estrogen and medroxyprogesterone lead to further increases in endothelin levels (38, 42, 43).

While the incidence of hypertension is undoubtedly higher in postmenopausal women compared to younger reproductive-aged women, the independent role that menopause plays in the incidence is contentious. Confounding factors are also known to increase with age, such as obesity, lipid levels and salt sensitivity, make it difficult to isolate the specific role of menopause (44–52).

### Combined Oral Contraceptives

Combined oral contraceptives (COC) are widely prescribed for birth control and many medical disorders in women, including menstrual bleeding disorders, ovarian cysts, and androgenization (53). The Nurses’ Health Study evaluated nearly 70,000 female nurses aged 25–42 and demonstrated that women taking oral contraceptives had a significantly higher risk of hypertension (44, 54). Samad et al. found that women with 6 or more years of oral contraceptive use were found to be at the greatest risk of developing hypertension (55). A personal history of pregnancy-induced hypertension, family history of hypertension, occult renal disease, obesity, age greater than 35 years, and increased duration of COC use are also found to increase susceptibility to hypertension while taking COCs (39, 56).

The mechanisms involved in oral contraceptive-induced hypertension are not well understood. RAAS and sympathetic activation may be involved, but whether this is due to the effects of estrogen, progesterone or an interaction between the two is unknown (54, 57). Early studies using high-dose estrogen of at least 50 mg and a progestin dose of 1–4 mg resulted in approximately 5% of women developing overt hypertension (58). Current COC formulations contain less than 20% of estrogen and progestin as previous preparations, but even these low dose COC formulations are associated with hypertension (44). Evidence suggests elevation in blood pressure due to COCs is reversible (39, 44, 54, 56). A controlled prospective study of 32 women who discontinued combination OCs after 1–3 years of use found that blood pressure returned to pretreatment levels within 3 months of discontinuation (55). If a patient remain hypertensive after 4 weeks of cession of COC, an evaluation for chronic hypertension should be performed (44). Although oral contraceptive-induced hypertension is reversible, COC use is contraindicated if a woman’s BP is >160/100 mm Hg as per WHO recommendations (55).

World Health Organization estimates that 151 million women use oral contraceptive pills worldwide (59). In South Asian countries like Pakistan and India, female sterilization and condoms are the primary methods of contraception; 4.1 and 7% of married women use COCs as their contraceptive method in India and Pakistan, respectively (60, 61). In Nepal and Sri Lanka, 5 and 5.9% of married women use COCs, respectively (62). Bangladesh is an exception where COCs are used by more than half of all modern contraceptive users; COCs and its high association with hypertension must be kept in mind when considering contraception choices (63).

### Polycystic Ovarian Syndrome

Polycystic ovarian syndrome (PCOS) is a common endocrine disease in women during reproductive age. WHO estimates 116 million girls are affected from PCOS worldwide (64). In India, experts report the prevalence of PCOS to be 10%, but there is no proper statistical data on PCOS prevalence in India yet (64). PCOS’s hallmarks include anovulation, androgen excess and insulin resistance (65). Several studies suggest that women with PCOS are at an increased risk of developing hypertension compared with the general population (66–73). Data also suggests that hyperandrogenism in women with PCOS may be associated with hypertension independent of obesity or insulin resistance (74–77). Other research suggests that insulin resistance and hyperinsulinemia cause hypertrophy of the vascular muscle wall and reduce vascular compliance by interfering with endothelium-dependent vasodilatation mechanisms (78). The Nurses’ Health Study found that women with irregular menstrual cycles had almost two times the risk for new diagnosis of hypertension, a risk that was not eliminated with adjustment for BMI (78). PCOS is a significant risk factor for preeclampsia as well. A meta-analysis demonstrated that pregnancy-induced hypertension and preeclampsia were both nearly 3.5 times more likely in women with PCOS (65). PCOS and a history of preeclampsia both lead to an increased risk of cardiovascular disease in women (44). These findings suggest that women with PCOS should be carefully monitored, screened for hypertension at an early age. Despite the paucity of data on the prevalence of PCOS in South Asian countries, PCOS is a major risk factor of hypertension and needs to be considered when working up hypertension in women.

### Pre-eclampsia

Hypertension in pregnancy has been associated with an increased risk of future hypertension and cardiovascular events (79–84). Preeclampsia is a pregnancy complication characterized by hypertension and proteinuria after 20 weeks of gestation, impacting 3–8% of all pregnancies (85). Not only are women with a history of preeclampsia three to four times more likely to develop chronic hypertension, they have twice the risk for cardiovascular disease (CVD) and stroke (80, 86). The onset of preeclampsia before 32 weeks is even more detrimental, making women five times more likely to develop CVD (44). Following up women with a history of preeclampsia for a minimum of 2 years, Sibai et al. found a substantially higher incidence of hypertension compared with normotensive controls (87). An Italian retrospective study found half of the participating women with a history of preeclampsia were hypertensive 10 years after delivery and one third were hypertensive after 5 years (88). These data together underscore the importance of close follow-up and patient education after preeclampsia-complicated pregnancies.

In South Asian countries, the incidence of preeclampsia is higher compared to countries with more developed healthcare systems. For example, compared with Sweden and the United States where rates of pre-eclampsia were found to be 3.6–4%, in a prospective population-analysis, Magee et al. reported the incidence of hypertension in pregnancy in India and Pakistan to be 9.3 and 10.3%, respectively (89–91). Another study reported the incidence in Pakistan to be as high as 19% (92). Multiple studies report higher incidences of pregnancy-induced hypertension and eclampsia amongst adolescent mothers (93). The widespread practice of adolescent marriages in South Asia may contribute to the alarming preeclampsia statistics; 45% of women of 20–24 years report being married before the age of 18 with 17% married before the age of 15 (94). Bangladesh has the highest rates of child marriage in Asia; 50% of young women were married before the age of 18, and nearly 5 in 10 child brides gave birth before the age of 18 (95). In Bangladesh, pre-eclampsia and its related conditions are responsible for about 20% of all maternal deaths (96). Furthermore, women with pregnancy induced hypertension have a higher risk of developing essential hypertension later on in life (97). It is imperative for clinicians to stay on high alert when dealing with adolescent pregnancies and employ primary prevention strategies targeting essential hypertension as soon as PIH is identified. At the time of postnatal follow ups, women with a history of pregnancy-induced hypertension (PIH) or preeclampsia should be counseled of their increased risk for essential hypertension and encouraged to get their blood pressure screened frequently.

Studies indicate endothelial dysfunction plays a central role in the pathogenesis of preeclampsia and persists in women postpartum. This remaining damage increases the risk of developing CVD and hypertension (85, 98). Additionally, endothelial dysfunction correlates with higher levels of coronary calcium content which is associated with acute coronary events. An alternative explanation is that preeclampsia itself induces irreversible vascular and metabolic changes that may increase the overall risk for hypertension and CVD (98). Some studies suggest preeclampsia could be a marker for pre-existing CHD risk instead of an independent risk factor (99). The metabolic stress of pregnancy may simply be unmasking pre-existing predisposition to CVD which presents as preeclampsia. The recent guidelines of the American Heart Association acknowledge the significance of hypertension in pregnancy in prediction of female cardiovascular disease- cardiovascular risk should be evaluated as soon as 6 months post-delivery after a preeclampsia- complicated pregnancy (44).

## Complications of Hypertension

**FIGURE 2:**
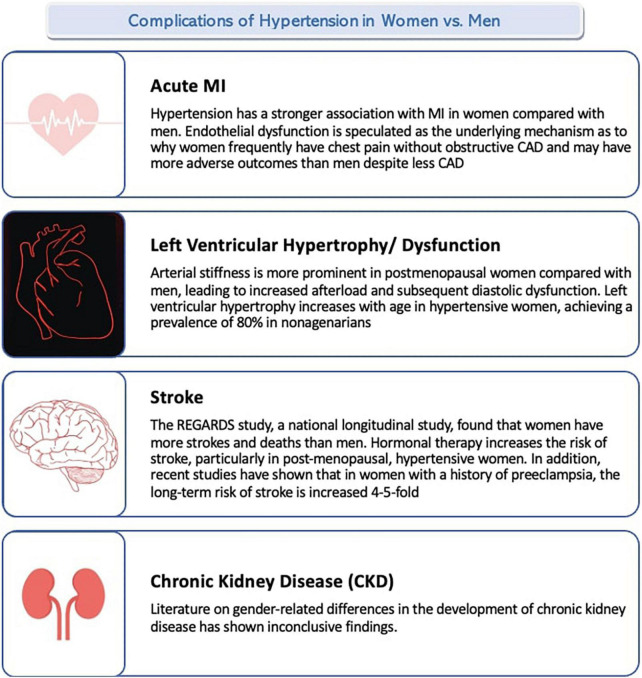
Complications of hypertension in women vs. men.

There are several complications of hypertension. The major ones include acute myocardial infarction, stroke, heart failure, and chronic kidney disease. Figure 2 summarizes the complications of hypertension in women vs. men.

### Acute Myocardial Infarction

Chronic hypertension is a well-known risk factor for myocardial infarction (MI), with a population attributable risk of 36%, indicating that the risk of MI could be decreased by 36% if hypertension is eliminated (100). Hypertension has a stronger association with MI in women compared with men (101). Pre-existing hypertension is associated with increased rates of death and morbid events; women in particular are more likely to die than men in the 12 months following acute myocardial infarction (AMI) (100). Furthermore, women with a systolic blood pressure >185 mmHg have thrice the risk of cardiac death compared with women with pressures <185 mmHg (102). There may be sex differences in the pathophysiology of AMI; in patients pooled from 11 independent ACS clinical trials, women with AMI had more non-obstructive coronary artery disease (CAD) than men (15 vs. 8%, respectively) (103). Endothelial dysfunction, as discussed earlier, is speculated as the underlying mechanism as to why women frequently have chest pain without obstructive CAD and may have more adverse outcomes than men despite less CAD. The higher burden of cardiovascular heart disease (CHD) in South Asians compared with other ethnicities has been frequently studied. South Asia accounts for 25% of the world’s population yet claims 60% of the global burden of heart disease (104). The INTERHEART study signified that myocardial infarctions occur approximately 10 years earlier in South Asian countries than in other regions and reported that South Asian migrants living elsewhere were prone to premature CHD (104).

### Left Ventricular Hypertrophy/Dysfunction

The most widely accepted model of hypertensive heart failure (HF) includes chronic pressure overload which leads to the development of left ventricular hypertrophy (LVH). History of hypertension is correlated to a higher incidence of morbid events and fatality during the early period after an acute myocardial infarction (AMI) and exacerbates the long-term progression of AMI by LV dysfunction and/or heart failure (105). A recent review of 26 studies with more than 12,000 patients showed that the prevalence of left ventricular hypertrophy was 16% in women and 24% in men with hypertension (106). On the contrary, a population-based study in Pakistan found women at an eleven-fold higher risk of developing LVH compared to men (107). The structural changes in left ventricular hypertrophy, assessed by echocardiogram, are different in hypertensive men and women. Hypertensive women are more likely to develop concentric hypertrophy, and men are more likely to develop eccentric hypertrophy (108). Left ventricular hypertrophy increases with age in hypertensive women, achieving a prevalence of 80% in nonagenarians (109). Furthermore, previous studies have proposed that women may have an inherent predisposition to develop LVH at any given pressure load (110–112). Data is not consistent regarding LV diastolic dysfunction. A report using magnetic resonance tissue phase mapping of myocardial motion showed that diastolic function was superior in women compared to men at a younger age. This association reversed as age increased and the loss of function became greater in women (113). Arterial stiffness is more prominent in postmenopausal women compared with men, leading to increased afterload and subsequent diastolic dysfunction (114). At present, the pathophysiology behind this differential age and gender effect are unclear and are likely to be multifaceted (114). Body mass index, cholesterol levels, and diabetes clarify for only 50% of the age-related increase in cardiovascular morbidity and mortality among women (115). Hence, additional factors should be considered in the high prevalence of CVD in older women.

### Stroke

Hypertension is the primary risk factor for stroke. The Reasons for Geographic and Racial Differences in Stroke (REGARDS) study, a national longitudinal study, found that women have more strokes and deaths than men (116). In a literature review of 18 studies, Gorgui et al. evaluated blood pressure and the risk of stroke in women; they found a 10 mmHg increase in systolic BP associated with a 38% increased stroke risk in women (117). Hormonal therapy increases the risk of stroke, particularly in post-menopausal, hypertensive women (117). In addition, recent studies have shown that in women with a history of preeclampsia, the long-term risk of stroke is increased 4–5-fold (118). Hypertensive disorders of pregnancy also increase stroke risk. In a multiethnic cohort study, Eastwood et al. found South Asians at twice the risk of suffering a stroke than Europeans (119). Furthermore, South Asians had a more adverse blood pressure profile compared to Europeans.

### Chronic Kidney Disease

Literature on gender-related differences in the development of chronic kidney disease has shown inconclusive findings. One theory suggests that estrogen exerts a protective role on renal function thus postmenopausal women experience a more rapid decline in renal function than men (120). In some cross-sectional studies, microalbuminuria was found to be more common in hypertensive men whereas other studies found no gender-related differences (121–124). Palatini et al. found microalbuminuria to be more likely to develop in hypertensive premenopausal women than in men of similar age (125). In a study from the Control of Blood Pressure and Risk Attenuation-Bangladesh, Pakistan and Sri Lanka (COBRA-BPS) trial, the prevalence of albuminuria was higher in woman than in men across all age ranges (126).

## Treatment

### Lifestyle Modifications

Lifestyle modification is the first line of antihypertensive treatment regardless of gender (127). A healthy lifestyle, including diet and exercise, can significantly delay the onset of hypertension, reducing cardiovascular risk, and further enhance the effects of pharmacological treatment. Studies show reduced alcohol intake effectively lowers blood pressure in both hypertensive and normotensive individuals and may help prevent the development of hypertension (128). The Nurses’ Health Study found obesity to be the most powerful predictor of hypertension with an attributable risk of 40%; in the Framingham Offspring Study, hypertension in 78% in men and 65% in women was attributable to obesity (86, 129). Obesity is more common in South Asian women than men; it is a significant risk factor that should be considered for women (130). Obesity at a young age is a strong indicator for future hypertension (131). Increased body fat is associated with impaired endothelium-dependent vasodilation, counteracting the protective role estrogen plays in premenopausal women (132). Data from four prospective cohort studies examining subjects from adolescence to early middle age demonstrated that being obese continuously or acquiring obesity was associated with a relative risk of 2.7 for developing hypertension. For those who became non-obese as adults, the risk of developing hypertension was similar to those who had a normal body mass index (BMI) from childhood to adulthood. Hence, it is essential for women to take interventions throughout their lives, from their adolescent years to postmenopausal, to maintain a normal BMI. Inadequate physical activity is one of the most important modifiable risk factors for hypertension (133). Whilst the effect of physical activity on the hypertensive heart remains limited, studies report that high-intensity interval training has cardioprotective effects. Exercise overall appears to have a positive effect on hypertensive heart remodeling with paradoxical regression of LVH (134). According to the WHO, people should have at least “600 metabolic equivalent minutes (MET minutes)” of physical activity per week- equivalent to 150 min of brisk walking per week. South Asia, the Middle East and Africa all have the highest prevalence of low physical activity of 21.6% (135). In South Asian countries, women are less active than men (136). Qualitative and quantitative evidence indicate that South Asian women have inadequate levels of physical activity (137). Physical inactivity in women stems from cultural and societal gender norms. Efforts to increase awareness of the importance of physical activity and address societal restraints for women must be made to encourage physical activity in women, thereby addressing a significant risk of hypertension in the population. Improving the gender gap in physical activity could have a substantial impact on overall population health.

### Pharmacological Treatment

Historically, hypertension in women has received significantly less attention compared to men (138). In general, women are excluded from clinical research and trials; they comprise a mere one-third of study populations for cardiovascular drug trials (139). For example, the Multiple Risk Factor Intervention Trial for the Prevention of Coronary Heart Disease (MRFIT), a national study with 12,866 participants, included no women (140). For women in general, and particularly older women, the blood pressure threshold for initiating drug treatment, target goals, and which drugs and drug combinations are most effective for reducing CV events are not conclusive. However, the 2017 ACC/AHA hypertension guidelines state there is no evidence that these issues differ for women and therefore, these guidelines recommend the same approach for treating both hypertensive men and women (141). The Blood Pressure Lowering Treatment Trialists’ Collaboration’s meta-analysis including 31 randomized trials with around 100,000 men and 90,000 women with hypertension found substantial evidence that the efficacy of antihypertensive drugs is similar in men and women (138). There are many classes of antihypertensive drugs; those that have been shown to reduce clinical events should be preferentially used. The primary drugs used in the treatment of hypertension include thiazide diuretics, ACE inhibitors, angiotensin receptor blockers (ARBs), and calcium-channel blockers (CCBs) (142–144).

Many factors can, however, influence the choice of antihypertensive medications. Studies have described differences in antihypertensive drug prescription and use in hypertensive men vs. women. Pharmacodynamic differences have been noted with amlodipine, a calcium channel blocker; a multicentric study with 1,000 patients found a greater blood pressure response in women as well as a higher percentage of women achieving blood pressure target goal (145). Comorbidities in women may influence the choice of antihypertensive treatment toward diuretics. Thiazide diuretics may have a positive effect on osteoporosis in postmenopausal women due to reduction of urinary calcium excretion; its use is associated with a decreased risk of hip fractures (57, 146, 147). In addition, studies suggest thiazides and calcium channel blockers may be beneficial in reducing the risk of stroke in elderly women compared to ACE inhibitors (148, 149).

A meta-analysis of data from 46 population-based studies in 22 countries including 123,143 men and 164,858 women aged 20–59 years showed that women with hypertension were 1.33 fold more likely to be treated with medication and were more commonly prescribed diuretics while more men used beta blockers, ACE inhibitors, and calcium channel blockers (146). An analysis of more than 12,000 visits in primary care facilities showed that diuretics were used in 20.9% of women vs. 16.9% in men, while ACE-inhibitors were used in 28.7% of men vs. 20.9% in women (150). Women may experience more adverse effects of antihypertensive therapy than men which can also impact the choice for therapy (57, 146). Lewis et al. found adverse effects twice more frequent in women than men, suffering more from coughing induced by ACE inhibitors (151).

All antihypertensive drugs cross the placenta; there is a general paucity of data for selection of hypertensive medications during pregnancy due to lack of trials observing the efficacy and safety of antihypertensive drugs (99). In women of reproductive age, selection of antihypertensive medication must be made keeping into consideration medications which are contraindicated during pregnancy including ACE inhibitors, ARBs, or direct renin inhibitors (44).

Severe fetopathy has been well documented in pregnant women with exposure to ACE inhibitors or ARBs, including death, end-stage renal disease, intrauterine growth restriction, oligohydramnios, and severe cerebral and pulmonary complications. Both of these drug classes are contraindicated in reproductive-age women in the absence of effective contraception (152, 153). According to the 2020 International Society of Hypertension Guidelines, the first choices for medication during pregnancy are methyldopa, beta-blockers (labetalol), and dihydropyridine-calcium channel blockers (DHP-CCBs) [nifedipine (not capsular), nicardipine] (127). Since all antihypertensives are secreted into breast milk in low concentrations, long acting calcium channel blockers are preferred; atenolol, propranolol, nifedipine should be avoided due to their high concentrations in milk (127).

The 2017 ACC/AHA hypertension guidelines have detailed algorithms for treatment recommendations based on BP thresholds and absolute CVD risk. Despite the reasonable notion that men and women are different, current hypertension guidelines do not recommend sex-specific strategies, and risk-assessment models do not consider risk factors specific to women. There is a comprehensive section for treatment of hypertension in pregnancy yet there are no recommendations for women of reproductive age who may be unaware of a pregnancy (127). Table 3 displays the current policies and plans for hypertension in South-Asian countries; currently none of the aforementioned countries have sex-specific guidelines for hypertension (154–161). Global health organizations and health agencies like the WHO must formulate clinical guidelines that are gender-specific from early adulthood and incorporate gender into their hypertension screening control programs. Furthermore, the guidelines endorsed by the WHO must include age ranges when recommending teratogenic drugs to women, discouraging their use by reproductive-aged women. Recommending teratogenic drugs like ACE inhibitors, ARBs, etc., to this age group could prove to be detrimental for both mother and child.

**TABLE 3:** Current policies and plans for hypertension in South-Asian countries.

Country	Existing national level policies/Plans	Gender- specific?
India	India Hypertension Control Initiative Indian Guidelines on Hypertension (IGH – IV)	No
Pakistan	Pakistan Hypertension League Guideline for detection, control, and management – 1998	No
Bangladesh	National Non-Communicable Disease Control (January 2017 – June 2022) National Guidelines for Management of Hypertension in Bangladesh	No
Nepal	Multisectoral Action Plan for the Prevention and Control of Non-Communicable Diseases (2014–2020)	No
Sri Lanka	National multisectoral action plan for the prevention and control of non-communicable diseases (2016–2020) CCP Hypertension Guidelines 2016	No
Bhutan	The multisectoral national action plan for the prevention and control of non-communicable diseases (2015–2020)	No
Afghanistan	Not available	–
Maldives	Not available	–

*Recommendations: Global health organizations (CDC, WHO, etc.) should incorporate gender into their hypertension screening control programs, risk assessment models and clinical guidelines for hypertension. Routinely screen women for hypertension across all medical specialties, particularly those women at increased risk for cardiovascular disease. Educate physicians and community health workers in South Asian countries on the gender-specific differences and management of hypertension. Prioritize community outreach and education messages that address hypertension for primary prevention of cardiovascular disease. Increase public awareness of hypertension as a serious risk in both men and women in order to improve the prevalence and treatment of hypertension worldwide. Educate communities on the importance of physical activity as primary prevention for hypertension.*

It is important to note that the importance of sex and gender has been recognized in clinical studies. A recent analysis of National Heart, Lung, and Blood Institute (NHLBI) funded research shows spending increased from $0.5 million in 1991 to $18.3 million in 2014 in research into sex differences in hypertension (162). However, a number of knowledge gaps still need to be filled. Further research is warranted to identify optimal sex-specific treatment options that will improve the control of hypertension and decrease the risk of subsequent cardiovascular disease in both genders.

## Awareness and Adherence

Medical adherence starts with awareness. Knowledge and awareness of hypertension must be improved in order to increase medication adherence and optimum blood pressure control. An American study reviewed control rates of hypertension among hypertensive men and women from 2003 to 2004 through 2011 to 2012 (163). They found awareness of hypertension increased in both men and women during this time period, with the greatest increase in awareness reported in women. National estimates of hypertension awareness in India is 44.7%, respectively, with male sex associated with decreased awareness (164). In rural central Punjab, Pakistan, a cross-sectional study similarly found male sex to be inversely associated with awareness of hypertension (165). 62.3% of patients with hypertension were aware of having high blood pressure. These results suggest improvement in awareness of hypertension compared to the 1990–1994 National Health Survey of Pakistan in which awareness of hypertension was 15.4% in men and 36% in women (165). However, further studies are needed to determine hypertension awareness throughout South Asian countries.

Public education is crucial to increase awareness about hypertension and promote blood pressure control in communities. Establishing community-based health education programs may improve regional hypertension rates and health outcomes in hypertensive patients. In a randomized controlled trial, Jafar et al. delineates how simple home health education by trained community health workers significantly reduced the expected increase in blood pressure with age in children and young adults in Pakistan (166). Developing and leveraging community outreach and education messages that address hypertension should be prioritized for primary prevention of cardiovascular disease.

Adherence is a well-recognized factor affecting hypertension control, involving patient’s regular use of medications, adherence to a modified diet plan and lifestyle changes (167, 168). Multiple studies have reported that a high percentage of hypertensive South Asian patients (33–67.6%) remain non-adherent to their medications (21). A population-based cohort study found, after adjustment for patient and clinical factors, South Asian and Chinese individuals had significantly lower adherence compared with White individuals (169). Although gender differences in medication adherence have not been specifically examined, medication adherence overall is suboptimal among South Asians.

Non-adherence to medications in general has been found to be a problem in women in South Asia, including India, Pakistan, Bangladesh, etc. With patriarchy embedded in South Asian culture, women face pervasive gender discrimination in all aspects of life, especially healthcare (170). Given much higher social, cultural, and economic barriers compared to men, gaining access to medical treatment is itself a uniquely challenging task for women residing in South Asian countries. With limited funds, purchasing medication for women is low priority on the list of expenses. Women ultimately defer to their husbands; these social priorities inherent in South Asian culture make women’s adherence to treatment extremely difficult (171). In a qualitative study, Kalra et al. delineates how South Asian women, especially those who are older, felt addressing their own health needs was not consistent with their primary role of dedicating themselves to the family (22).

## Summary and Implications

In summary, hypertension is one of the most important risk factors for cardiovascular disease. The pathophysiology of essential hypertension is still being delineated in both men and women; there are multiple sex specific factors in association with the development of hypertension in women, including age, combined oral contraceptives (COCs), polycystic ovarian syndrome (PCOS), preeclampsia, etc. There are several sex specific considerations in antihypertensives drug choices. Despite the magnitude of its negative impact on cardiovascular outcomes, treatment and control of hypertension remain suboptimal in women. Medical treatment and adherence is uniquely challenging for South Asian women due to a variety of socio-cultural-economic factors.

It is imperative to create integrative health systems which engage clinicians, healthcare workers, and patients in recognizing the differences between hypertension in men and women, requiring collaboration at local, national and global levels (86). Despite studies demonstrating the differences between the sexes regarding risk factors, pathophysiology, complications, treatment of hypertension, sex-specific hypertension guidelines are yet to be developed. To reduce hypertension rates and subsequent cardiovascular disease in both men and women, global health organizations such as the CDC and WHO must incorporate gender into their hypertension screening control programs, build risk assessment models which consider risk factors specific to women and formulate sex-specific clinical guidelines for hypertension. Despite an increase in spending in research into sex differences in hypertension, more studies are needed to identify sex-specific treatment options and develop evidence-based strategies to reduce hypertension-related complications and mortality.

**FIGURE 3:**
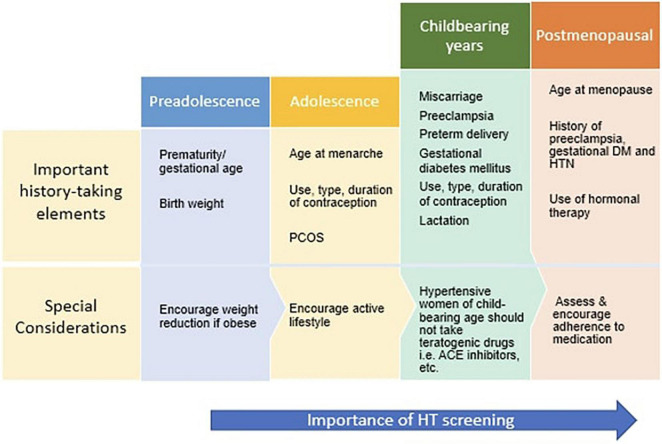
Considerations along the life cycle of a woman.

Medical professionals across all specialties should be encouraged to screen women routinely for hypertension, particularly those at increased risk for cardiovascular disease and postmenopausal women. Figure 3 displays the pertinent history taking elements to be addressed along the lifecycle of a woman and special considerations for each age group. Physicians and community health workers in South Asian countries should be educated on the gender-specific differences of hypertension and advised to employ primary prevention strategies targeting essential hypertension as soon as PIH is identified. This will allow for prompt identification and referral of high-risk women to tertiary care centers for appropriate treatment.

The differences in hypertension between men and women have implications not only for patients, but for the general public as well. Local community efforts must be made to increase public awareness of hypertension as a serious risk in both men and women in order to improve the prevalence and treatment of hypertension worldwide. Developing and leveraging community outreach and education messages that address hypertension should be prioritized for primary prevention of cardiovascular disease. Communities must be educated on the importance of physical activity as primary prevention for hypertension and efforts should be made to address societal restraints for women to encourage physical activity in women. A global approach to education, screening, and gender-based treatment for hypertension is one of the most crucial takeaways of this narrative review.

## Author Contributions

ZS, FF, MJ, and AAb contributed to conception and design of the study. FF and AAb wrote the first draft of the manuscript. AAl, SV, TJ, and ZS revised the manuscript critically. All authors contributed to manuscript revision, read, and approved the submitted version.

## Conflict of Interest

The authors declare that the research was conducted in the absence of any commercial or financial relationships that could be construed as a potential conflict of interest.

## Publisher’s Note

All claims expressed in this article are solely those of the authors and do not necessarily represent those of their affiliated organizations, or those of the publisher, the editors and the reviewers. Any product that may be evaluated in this article, or claim that may be made by its manufacturer, is not guaranteed or endorsed by the publisher.
